# Evaluation of the Safety and Efficacy of Trastuzumab Emtansine (T-DM1) Biosimilar in Human Epidermal Growth Factor Receptor 2 (HER2)-Positive Breast Cancer: Findings From a Single-Center Retrospective Analysis in India

**DOI:** 10.7759/cureus.76061

**Published:** 2024-12-20

**Authors:** Kaushal Patel, Vipulkumar Thummar, Priya Mehta

**Affiliations:** 1 Medical Oncology, Sunshine Global Hospital, Surat, IND; 2 Medical Affairs, Zydus Lifesciences, Ahmedabad, IND

**Keywords:** her-2 positive breast cancer, overall survival, progression free survival, t-dm1 biosimilar, trastuzumab emtansine

## Abstract

Background

Human epidermal growth factor receptor 2 (HER2)-positive breast cancer is a highly aggressive subtype characterized by a high recurrence rate. Trastuzumab emtansine (T-DM1), an antibody-drug conjugate targeting HER2, has shown improved outcomes; however, its effectiveness in cases with brain metastases remains unclear. The T-DM1 biosimilar has emerged as a cost-effective treatment option.

Objectives

The primary objective was to assess the efficacy of the T-DM1 biosimilar by evaluating progression-free survival (PFS) and overall survival (OS). The secondary objective focused on analyzing the safety profile, including the incidence and characteristics of adverse events (AEs) during T-DM1 treatment.

Materials and methods

In this retrospective, single-center study, 30 HER2-positive breast cancer patients were treated with T-DM1 at a dose of 3.6 mg/kg, administered intravenously every 21 days. The primary outcomes measured were PFS, OS, time to treatment failure (TTF), complete response (CR), and partial response (PR). The secondary endpoint was the documentation of AEs.

Results

All patients, with a median age of 57 years, received prior HER2-targeted therapy, and five (16.6%) patients had undergone brain radiotherapy. CR was achieved in 20 (66.6%) patients, while 10 (33.3%) patients attained PR. PFS, OS, and TTF were approximately 14, 24, and 13 months, respectively. Common adverse events included elevated transaminases, anemia, and thrombocytopenia, the majority of which were controllable and reversible.

Conclusion

This study provides important insights into the efficacy and safety of the T-DM1 biosimilar. The findings indicate that T-DM1 is effective in enhancing OS and managing advanced HER2-positive breast cancer, including selective patients with brain metastases. The results highlight the T-DM1 biosimilar as a cost-effective treatment option with an expected safety and tolerability profile.

## Introduction

Human epidermal growth factor receptor 2 (HER2)-positive breast cancer is notably aggressive due to the overexpression and abnormal amplification of the *HER2* gene. This subtype constitutes about 20% of breast cancer cases globally, with a national prevalence ranging from 26% to 50%, predominantly affecting young women [1]. The recurrence rate is slightly higher compared to other breast cancer variants [2,3]. Overexpression of the HER2 protein results in uncontrolled tumor growth, leading to a poor prognosis. Metastatic patients often develop resistance to first-line treatments, including surgery, radiotherapy, hormonal therapy, or chemotherapy, necessitating the need for second-line treatments [4].

Trastuzumab, a monoclonal antibody targeting HER2, initially played a significant role in managing HER2-positive breast cancer. However, issues such as potential cardiotoxicity, disease recurrence, and failure to respond within a year of treatment have been observed [5]. These challenges have highlighted the need for new HER2-targeted therapies to improve patient outcomes [6].

T-DM1, also known as trastuzumab emtansine, is a notable HER2-specific targeted agent. It combines the cytotoxic molecule DM1 with trastuzumab, which targets and binds to the extracellular domain of HER2 receptors, thereby reducing gene expression. DM1 disrupts microtubule function, causing cell cycle arrest and cell death [7,8]. The conjugation of trastuzumab and emtansine is connected by a stabilized linker, which, upon binding to the HER2 receptor, releases emtansine into the cytoplasm. This disrupts microtubules, inhibits HER2 signaling, and leads to cell cycle arrest and apoptosis [9-11].

The high cost of T-DM1 limits patient access. T-DM1 biosimilar provides a more affordable option, demonstrating high similarity to the original T-DM1 in terms of quality, efficacy, and safety. These biosimilars undergo rigorous evaluation through analytical, pharmacokinetic, and clinical studies to ensure their similarity and secure regulatory approval. Biosimilars aim to reduce the financial burden associated with cancer treatment while maintaining therapeutic outcomes [4].

Controlled clinical trials are often seen as the gold standard for evidence generation. However, they can be affected by selection bias and may not fully capture the complexities of real-world clinical settings [12]. This retrospective study aims to present firsthand experiences of the efficacy and safety of a T-DM1 biosimilar in routine clinical practice in India, comparing our treatment outcomes to those from controlled clinical trials. By doing so, we seek to provide valuable insights that bridge the gap between trial-based evidence and the practical realities of everyday clinical care. This research was accepted and presented as a poster at the European Society for Medical Oncology (ESMO) Asia, Singapore, on December 2, 2023, as well as at the Indian Breast Cancer Meeting (IBM), Mumbai, India, on April 5, 2024 [13,14].

## Materials and methods

General study details

This retrospective, single-center, observational study included patients treated with the T-DM1 biosimilar, evaluated between May 2021 and December 2022. Ethical committee approval was obtained to retrieve patient data. The ethical guidelines established by the Declaration of Helsinki, the New Drugs and Clinical Trials Rules 2019 (NDCT 2019), and the International Council for Harmonisation-Good Clinical Practice (ICH-GCP) were followed throughout the study. The Institutional Ethics Committee (IEC) granted permission for a waiver of consent due to the retrospective nature of the study.

Study objective

The primary objective was to evaluate the efficacy of the T-DM1 biosimilar by assessing progression-free survival (PFS) and overall survival (OS). The secondary objective was to analyze the safety profile, including the incidence and nature of adverse events observed during treatment.

Participants

A total of 30 patients aged 18 to 65 years with HER2-positive breast cancer and an Eastern Cooperative Oncology Group (ECOG) performance score of 2 or lower who received T-DM1 were included [15].

Inclusion Criteria

Eligible participants were female patients with pathologically confirmed measurable invasive HER2-positive metastatic breast cancer. These patients had previously undergone chemotherapy for metastatic disease, including trastuzumab and a taxane, either as separate treatments or in combination. Additionally, patients who experienced disease recurrence during or within six months after completing adjuvant therapy were considered for inclusion in the study.

Exclusion Criteria

Patients with a prior history of trastuzumab emtansine treatment, serious cardiac arrhythmias requiring intervention, symptomatic chronic heart failure, severe or uncontrolled systemic diseases (such as significant cardiovascular or pulmonary conditions, uncontrolled hypertension, or unstable angina), pregnancy or lactation, active infections with HIV, hepatitis C, or hepatitis B, hypersensitivity to trastuzumab emtansine, or any other significant medical or psychiatric conditions were excluded from the analysis.

Study methodology

Patients received the T-DM1 biosimilar (UJVIRA™, Zydus Lifesciences, Ahmedabad, India) at the standard dosage of 3.6 mg/kg, administered intravenously every 21 days.

Efficacy Endpoints

Brain metastases were evaluated using contrast-enhanced MRI or CT scans, with radiologists assessing the presence of metastases and tumor response to T-DM1 therapy. Tumor responses were determined based on the Response Evaluation Criteria in Solid Tumors (RECIST), version 1.1 [16]. The clinical benefit rate (CBR) was calculated as the proportion of patients achieving either a Complete Response (CR) or a Partial Response (PR) lasting six months or more. Additionally, the time to treatment failure (TTF) was analyzed. PFS and OS were determined retrospectively from the date of the first T-DM1 dose to either disease progression, death, or the last follow-up. TTF was calculated from the initiation of T-DM1 therapy to treatment discontinuation for any reason, such as disease progression, treatment toxicity, financial concerns, or death. The reasons for treatment discontinuation were documented in the medical records.

Safety Endpoints

Safety data were retrospectively reviewed based on adverse event reports. These events were monitored and graded according to the National Cancer Institute Common Toxicity Criteria, version 4.0. The safety assessment focused on identifying and evaluating the severity of treatment-related toxicities, including both clinical and laboratory abnormalities. Any treatment interruptions, dose reductions, or discontinuations due to adverse events were also recorded and analyzed to ensure a comprehensive evaluation of the safety profile of T-DM1 therapy.

Statistical analysis

Patient characteristics and related data were summarized using descriptive statistics. PFS was defined as the interval from the initial dose of T-DM1 to either disease progression or death. OS was calculated from the first dose of T-DM1 to the time of death. Survival estimates were determined using the Kaplan-Meier method with IBM SPSS Statistics for Windows, Version 21 (Released 2012; IBM Corp., Armonk, New York). A P-value of less than 0.05 was considered statistically significant.

## Results

**Figure 1:**
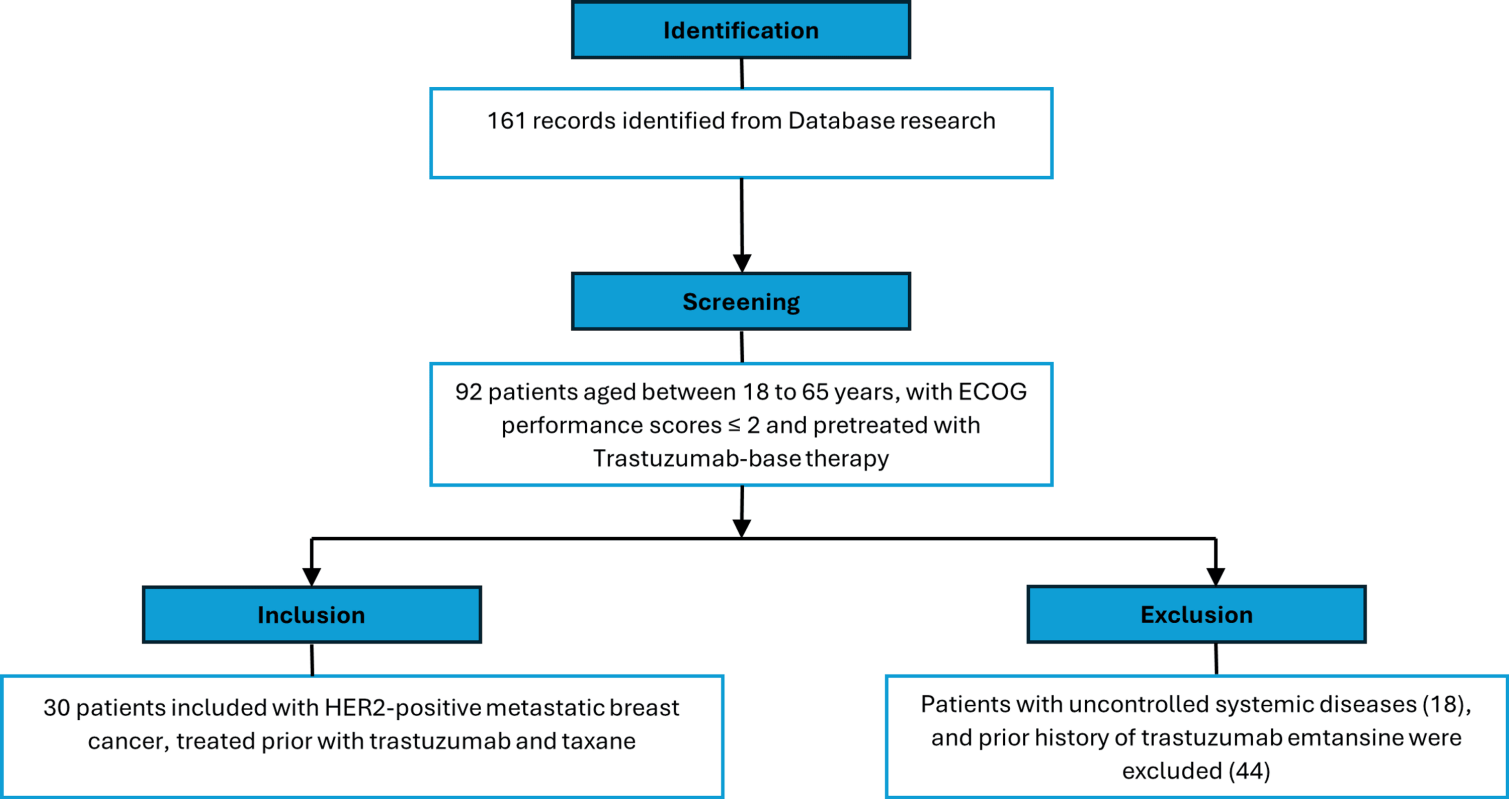
Flowchart for patient inclusion

In this retrospective observational study, 30 patients with HER2-positive breast cancer who met the inclusion criteria were selected for analysis (Figure 1).

The baseline characteristics of the study population are summarized in Table 1. The median age was 57 years (range: 42-75), with 26 (86.6%) patients being post-menopausal and an ECOG score of 1 in 17 (56.6%) patients. Additionally, 24 (80%) of the patients had ER− and PR− HER2+ receptor status, and all patients had visceral involvement. Brain metastases were observed in 5 (16.6%) of the patients. The study revealed that 19 (63.3%) patients had progressed to metastatic breast cancer following treatment for early breast cancer (EBC) or locally advanced breast cancer (LABC), while 11 (36.6%) patients were diagnosed with de novo metastatic breast cancer. Eighteen (60%) patients presented with two or more metastatic sites, and brain metastases were observed in 5 (16.6%) patients of the cohort (Table 1).

**Table 1:** Baseline characteristics of study population ER, estrogen response; PR, progesterone response; EBC, early breast cancer; ECOG, Eastern Cooperative Oncology Group; LABC, locally advanced breast cancer; mBC, metastatic breast cancer.

Characteristics	Values
Total patients	30
Age (years), median (range)	57 (42-75)
Weight (kg), median (range)	56.5 (45-67)
Other comorbidities	N (%)
Hypertension	3 (10)
Diabetes mellitus	2 (6.6)
Menopausal status	
Pre-menopausal	4 (13.3)
Post-menopausal	26 (86.6)
ECOG score	
0	9 (30)
1	17 (56.6)
2	4 (13.3)
Hormone receptor status	
ER+ and/or PR+ HER2+	6 (20)
ER− and PR− HER2+	24 (80)
Visceral involvement	
No	0
Yes	30 (100)
Non-visceral involvement	
No	28 (93.3)
Yes	2 (6.6)
Brain involvement	
No	25 (83.3)
Yes	5 (16.6)
Radiation therapy for brain metastasis	
No	25 (83.3)
Yes	5 (16.6)
Number of metastatic sites	
<2	10 (33.3)
≥2	18 (60)
Not reported	2 (6.7)
Stage at initial diagnosis	
1	1 (3.3)
2	14 (46.6)
3	4 (13.3)
4	9 (30)
Unknown	2 (6.6)
Disease status at current presentation	
De novo metastatic	11 (36.6)
EBC/LABC prior, now progressed to mBC	19 (63.3)

All patients had previously received trastuzumab-based therapy. Pertuzumab was administered to 13 (43.3%) of the participants, while 2 (6.6%) received Lapatinib and Capecitabine. Regarding T-DM1 biosimilar treatment, 25 (83.3%) of the patients received it as a second-line therapy, while 4 (13.3%) were treated with it as a third-line option. The majority of patients (20 (66.6%)) underwent 11-20 cycles of T-DM1 therapy. Dose reduction was not required for 27 (90%) of the patients (Table 2).

**Table 2:** Study subject distribution according to T-DM1 biosimilar treatment-related characteristics

Characteristics	N (%)
Treatment line for T-DM1 use	
1	1 (3.3)
2	25 (83.3)
3	4 (13.3)
Total number of cycles with T-DM1 biosimilar	
≤10	2 (6.6)
11-20	20 (66.6)
21-30	7 (23.3)
>30	1(3.3)
Dose reduction required	
No	27 (90.0)
Yes	3 (10.0)

**Figure 2:**
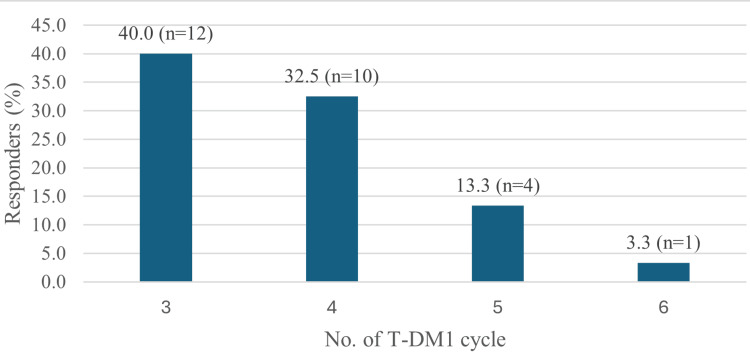
Best response achieved toward T-DM1 therapy (N=50)

The therapeutic response was noteworthy, with 20 (66.6%) of the patients achieving a CR and 10 (33.3%) showing a PR. Optimal responses described as best achievable outcome from treatment, such as complete or partial tumor reduction, were most commonly observed during the third (12 (40%)) and fourth (10 (32.5%)) cycles of treatment (Figure 2).

**Figure 3:**
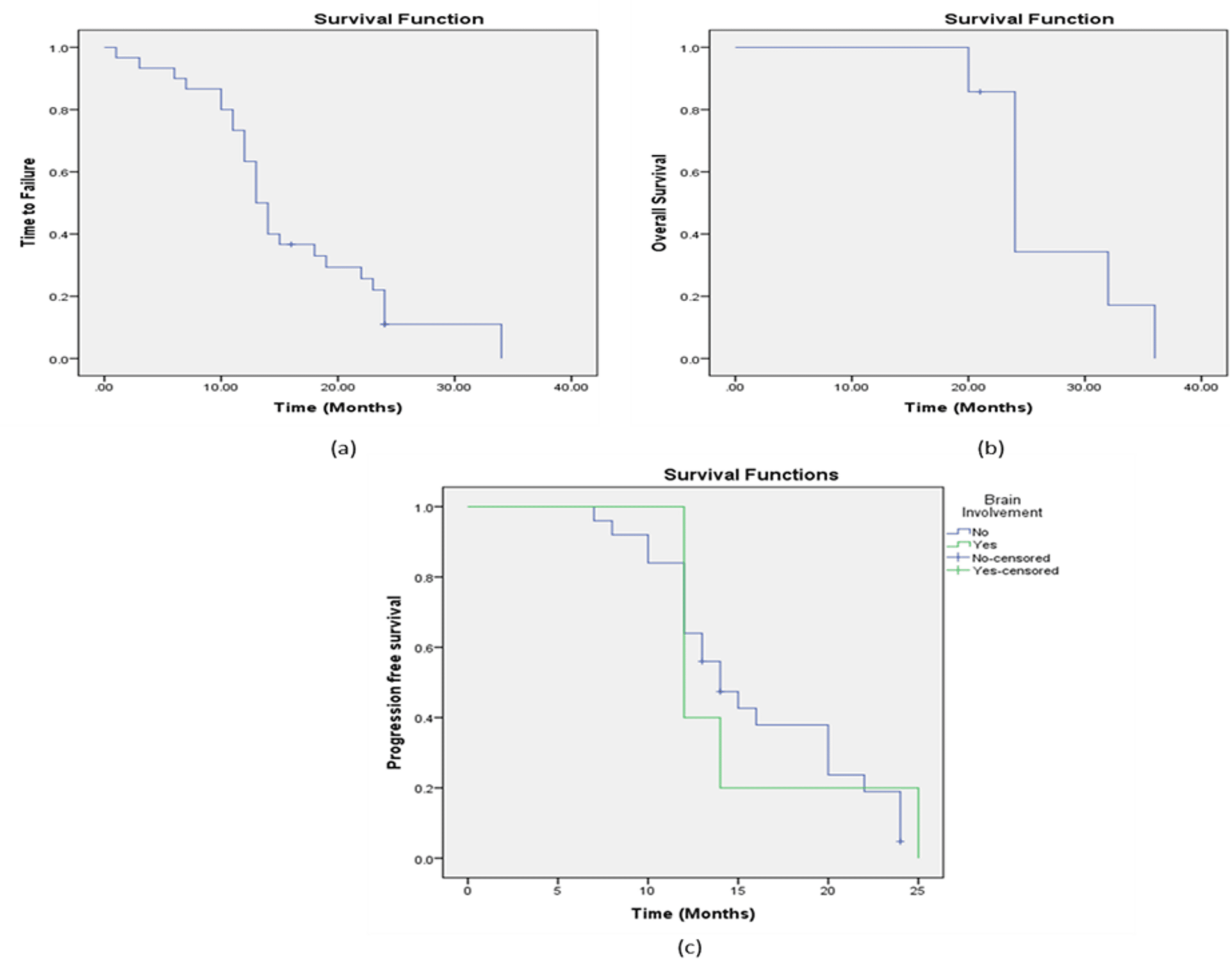
Clinical outcome in overall population treated with T-DM1 biosimilar: (a) Time to treatment failure in patients treated with T-DM1 biosimilar treatment. Median TTF = 13 months (95% CI: 11.47-14.53) (b) Overall survival in overall population treated with T-DM1 biosimilar. Median OS = 24 months (95% CI: 21.03-26.97). (c) PFS in patients without metastases of the central nervous system (CNS) (blue line) and in patients with CNS metastases (green line).

The median TTF for patients receiving T-DM1 therapy was 13 months (95% CI: 11.47-14.53), representing the median duration during which patients experienced disease control or a positive response to the treatment. The median OS for the entire population treated with T-DM1 therapy was approximately 24 months (95% CI: 21.03-26.97) (Figure 3a,b). The PFS rate for patients without central nervous system (CNS) metastases gradually decreased over time, with a notable decline around 20 months. In contrast, patients with CNS metastases showed a more rapid decline in PFS, with a significant drop observed around 10 months (Figure 3c).

Adverse events were predominantly mild to moderate, with no Grade 5 toxicities reported. The most common adverse events were elevated transaminases observed in 24 (80%) patients, thrombocytopenia observed in 29 (96.6%), and anemia observed in 8 (26.6%) patients. Severe adverse events were rare, with Grade 3 or 4 thrombocytopenia observed in 2 (6.6%) of the patients and reduced left ventricular ejection fraction (LVEF <50%) reported in 1 (3.3%) patient (Table 3).

**Table 3:** Frequency distribution of study population according to adverse events LVEF, left ventricular ejection fraction.

Type of Adverse Event	Grade 1	Grade 2	Grade 3	Grade 4	Total
Elevated transaminases	17	7	0	0	24 (80%)
Thrombocytopenia	6	21	1	1	29 (96.6%)
Anaemia	6	2	0	0	8 (26.6%)
Neutropenia	2	0	0	0	2 (6.6%)
LVEF <50%	0	0	1	0	1 (3.3%)
Hyperglycemia	0	1	0	0	1 (3.3%)
Fatigue	1	1	0	0	2 (6.6%)

## Discussion

This study offers valuable insights into the demographics, treatment outcomes, and safety profile of T-DM1 therapy in patients with advanced HER2-positive breast cancer. The predominance of post-menopausal patients aligns with the typical age distribution observed in breast cancer demographics [17]. A notable finding is that 63.3% of patients had progressed to metastatic breast cancer, highlighting the persistent challenge of disease progression despite prior treatments. According to the study, 37% of patients were initially diagnosed at stage IV, showcasing the critical need for improved early detection strategies to prevent advanced disease presentations [18].

Prior to receiving T-DM1, patients in our study had undergone various adjuvant or neoadjuvant therapies. All patients had been treated with trastuzumab, a HER2-targeted therapy, underscoring its established role in HER2-positive breast cancer treatment. These findings are consistent with the EMILIA and TH3RESA trials, which demonstrated the efficacy of T-DM1 in patients previously treated with HER2-targeted therapies [19,20]. In the EMILIA trial, T-DM1 was compared to lapatinib plus capecitabine in previously treated HER2-positive metastatic breast cancer patients. T-DM1 significantly improved PFS (9.6 vs. 6.4 months) and OS (30.9 vs. 25.1 months) over the lapatinib-capecitabine combination, establishing T-DM1 as a preferred option in this setting [19]. Similarly, the TH3RESA trial assessed T-DM1's efficacy in patients with advanced HER2-positive breast cancer who had received two or more prior HER2-targeted therapies. The study found that T-DM1 extended median OS by nearly seven months compared to the treatment of the physician's choice, underscoring its benefit in heavily pretreated populations [20].

In our study, we observed a higher objective response rate and PFS, which may be attributed to the high proportion of HER2-enriched patients and the smaller sample size. The OS depends on the subsequent therapies available to patients in real-world settings. We noted that patients with brain metastases had an OS of 24 months following T-DM1 therapy, comparable to those without brain metastases. This suggests that T-DM1 may effectively manage brain metastases, a condition typically associated with poorer outcomes. Supporting this observation, the KAMILLA trial reported that T-DM1 exhibited activity and was well tolerated in patients with HER2-positive metastatic breast cancer and brain metastases, with a median PFS of 5.5 months and an OS of 18.9 months in this subgroup [21]. Typically, brain metastases are linked to reduced survival times [22]. However, the comparable overall survival between patients with and without brain metastases indicates that T-DM1 therapy might effectively control the disease, even in the presence of brain metastases, or due to an underpowered comparison of these two cohorts. This could be important for enhancing survival in this particular patient group.

In terms of safety, most patients experienced mild to moderate adverse events (Grade 1 and 2), though a few encountered more severe adverse events such as thrombocytopenia and reduced left ventricular ejection fraction. These severe events can be managed with supportive therapy and dose adjustments. This safety profile aligns with findings from the KATHERINE trial, which also evaluated T-DM1’s safety [23].

Despite the significant findings, the retrospective design may introduce inherent biases, as patient selection and data collection were not randomized or controlled. Additionally, being conducted at a single center may limit the generalizability of the results to a broader patient population. Furthermore, the relatively small sample size might not fully capture the variation in patient responses to T-DM1 therapy. Nevertheless, it provides valuable insights into the use of the T-DM1 biosimilar in metastatic breast cancer patients, including those with brain metastases. This contributes to the broader understanding of T-DM1 therapy in managing advanced HER2-positive breast cancer.

## Conclusions

The study concluded that T-DM1 effectively enhances PFS and OS in patients with advanced HER2-positive breast cancer. Furthermore, the results indicate that the T-DM1 biosimilar offers an accessible treatment option with an expected safety and tolerability profile. These findings support the use of T-DM1 in real-world scenarios for metastatic breast cancer patients, including selective patients with brain metastases.

## Appendices

**Table 4:** Putting in perspective T-DM1: trastuzumab emtansine, HER2+: human epidermal growth factor receptor 2-positive, PFS: progression-free survival, OS: overall survival.

Putting in Perspective	
Central question	What gaps in knowledge exist between clinical trial results and routine clinical practice for T-DM1 biosimilar in India?
	How effective is the T-DM1 biosimilar in real-world settings for HER2+ breast cancer patients with brain metastases?
Key findings	Complete response achieved in 20 patients (66.63%)
	Partial response achieved in 10 patients (33.33%)
	Median progression-free survival (PFS) of nearly 14 months
	Median overall survival (OS) of approximately 24 months
	Common adverse events: elevated transaminases, anemia, thrombocytopenia; manageable and reversible
Impact	T-DM1 biosimilar demonstrates effectiveness and safety in a real-world clinical setting, bridging the gap between trial-based evidence and everyday practice
	Provides a cost-effective treatment option for Indian patients with HER2+ breast cancer, particularly benefiting those with advanced disease and selective patients with brain metastasis
